# Increased airway iron parameters and risk for exacerbation in COPD: an analysis from SPIROMICS

**DOI:** 10.1038/s41598-020-67047-w

**Published:** 2020-06-29

**Authors:** William Z. Zhang, Clara Oromendia, Sarah Ann Kikkers, James J. Butler, Sarah O’Beirne, Kihwan Kim, Wanda K. O’Neal, Christine M. Freeman, Stephanie A. Christenson, Stephen P. Peters, J. Michael Wells, Claire Doerschuk, Nirupama Putcha, Igor Barjaktarevic, Prescott G. Woodruff, Christopher B. Cooper, Russell P. Bowler, Alejandro P. Comellas, Gerard J. Criner, Robert Paine, Nadia N. Hansel, Meilan K. Han, Ronald G. Crystal, Robert J. Kaner, Karla V. Ballman, Jeffrey L. Curtis, Fernando J. Martinez, Suzanne M. Cloonan

**Affiliations:** 1 Joan and Sanford I. Weill Department of Medicine, Division of Pulmonary and Critical Care Medicine, Weill Cornell Medicine, New York, USA; 2 New York-Presbyterian Hospital, New York, New York USA; 3 Department of Population Health Sciences, Division of Biostatistics, Weill Cornell Medicine, New York, New York USA; 4 Department of Genetic Medicine, Weill Cornell Medicine, New York, New York USA; 5 University of North Carolina Marsico Lung Institute, Chapel Hill, North Carolina USA; 6 Pulmonary and Critical Care Medicine Division, Department of Internal Medicine, University of Michigan Health System, Ann Arbor, Michigan USA; 7 Veterans Affairs Ann Arbor Healthcare System, Ann Arbor, Michigan USA; 8 University of California at San Francisco, San Francisco, California USA; 9 Wake Forest School of Medicine, Winston-Salem, North Carolina USA; 10 Division of Pulmonary, Allergy and Critical Care Medicine, University of Alabama at Birmingham, Birmingham, Alabama UK; 11 Johns Hopkins University School of Medicine, Baltimore, Maryland USA; 12 Division of Pulmonary and Critical Care Medicine, University of California Los Angeles Medical Center, Los Angeles, California USA; 13 University of Colorado School of Medicine, Aurora, Colorado USA; 14 National Jewish Health, Denver, Colorado USA; 15 Division of Pulmonary and Critical Care, University of Iowa, Iowa City, Iowa USA; 16 Department of Pulmonary & Critical Care Medicine, Temple University, Philadelphia, Pennsylvania USA; 17 Section of Pulmonary and Critical Care Medicine, Salt Lake City Department of Veterans Affairs Medical Center, Salt Lake City, Utah USA; 18 School of Medicine, Trinity Biomedical Sciences Institute and Tallaght University Hospital, Trinity College Dublin, Trinity, Ireland; 19 SPIROMICS investigators, Collaborative Studies Coordinating Center, Department of Biostatistics Gillings School of Global Public Health, University of North Carolina at Chapel Hill 123 W. Franklin Street Suite 450, Chapel Hill, NC 27516 USA

**Keywords:** Respiratory tract diseases, Iron

## Abstract

Levels of iron and iron-related proteins including ferritin are higher in the lung tissue and lavage fluid of individuals with chronic obstructive pulmonary disease (COPD), when compared to healthy controls. Whether more iron in the extracellular milieu of the lung associates with distinct clinical phenotypes of COPD, including increased exacerbation susceptibility, is unknown. We measured iron and ferritin levels in the bronchoalveolar lavage fluid (BALF) of participants enrolled in the SubPopulations and InteRmediate Outcome Measures In COPD (SPIROMICS) bronchoscopy sub-study (n = 195). BALF Iron parameters were compared to systemic markers of iron availability and tested for association with FEV_1_ % predicted and exacerbation frequency. Exacerbations were modelled using a zero-inflated negative binomial model using age, sex, smoking, and FEV_1_ % predicted as clinical covariates. BALF iron and ferritin were higher in participants with COPD and in smokers without COPD when compared to non-smoker control participants but did not correlate with systemic iron markers. BALF ferritin and iron were elevated in participants who had COPD exacerbations, with a 2-fold increase in BALF ferritin and iron conveying a 24% and 2-fold increase in exacerbation risk, respectively. Similar associations were not observed with plasma ferritin. Increased airway iron levels may be representative of a distinct pathobiological phenomenon that results in more frequent COPD exacerbation events, contributing to disease progression in these individuals.

## Introduction

Chronic obstructive pulmonary disease (COPD) is a leading cause of mortality and morbidity worldwide, resulting in almost 3 million deaths globally in 2016 and the loss of over 47 million potential life-years^1^. A significant portion of this burden of disease presents as exacerbation events, episodic surges of respiratory symptoms accompanied by a more rapid decline in lung function and higher mortality^2,3^. These exacerbations are largely attributed to recurrent acute infection, with host factors also playing a fundamental role^4^. Identifying and understanding host susceptibility risk factors that contribute to repeated exacerbations is imperative to understanding and treating COPD.

Although the overwhelming risk factor in the industrialized world for COPD development is tobacco smoking, genome wide association studies suggest a pathogenic role for abnormal iron homeostasis^5^. We recently demonstrated that a major iron metabolism protein, iron regulatory protein *2 (IRP2)*, drives lung inflammation and injury in a murine model of COPD^5,6^. In the lung, iron is found in both unbound and protein-bound forms, and several of the most abundant proteins in lung tissue and bronchoalveolar lavage fluid (BALF) bind to and regulate iron^7–9^. One such protein is ferritin, an octahedral polymeric shell composed of light chain (FTL) and heavy chain (FTH) subunits that stores ferric (Fe^3+^) iron atoms in a soluble, non-toxic form^10^. Previous studies have demonstrated release of ferritin from iron-loaded alveolar macrophages (AMs) in smokers, and AM ferritin mRNA levels are increased in active smokers and correlate with airflow limitation in COPD patients^11,12^. Furthermore, total levels of non-heme iron and of other iron-binding molecules including lipocalin-2 and lactoferrin, are increased in lung tissue, sputum, BALF, and AMs of COPD patients, relative to non-smokers^9,11–21^. Conversely, there is also ample evidence for iron deficiency in COPD, and anaemia in COPD is associated with worse patient outcomes, including mortality^22,23^. The biological relevance of such observations remains to be elucidated; however, these data strongly support a local iron overload signature in the extracellular milieu of the lung in COPD that is distinctive to systemic iron handling, which is intriguing as mainstream cigarette smoke contains little iron^24,25^.

Whether overloaded or deficient, abnormal iron stores may have important immunologic and microbiologic implications, and could provide insights into COPD pathogenesis and progression^26^. However, to our knowledge, iron parameters have not been examined for association with clinical phenotypes or outcomes (including exacerbation frequency) in a large, well-characterized COPD cohort. In this study, we measured BALF ferritin and iron levels in participants enrolled in the extensively characterized SubPopulations and InteRmediate Outcome Measures In COPD (SPIROMICS) cohort. We demonstrate that BALF ferritin and iron levels, but not systemic iron parameters, are associated with clinical measures of disease activity, most importantly exacerbation frequency. These findings support a hypothesis whereby higher BALF iron levels, reflective of iron overload in the lung microenvironment, may represent a novel pathobiological endotype in COPD.

## Results

### Bronchoscopy sub-study participants

SPIROMICS (ClinicalTrials.gov NCT01969344T4) is an on-going longitudinal multicentre observational study that recruited 2981 subjects, 40 to 80 years of age, including never-smokers (≤1 pack-year of tobacco-smoking history), current or former smokers (ever-smokers, ≥20 pack-years) without airflow obstruction, and ever-smokers with airflow obstruction^27^. A subgroup of subjects (n = 215) with post-bronchodilator FEV_1_ > 30% predicted and without an exacerbation in the prior six weeks were further enrolled in a bronchoscopy sub-study. The characteristics of participants of the bronchoscopy sub-study, in relation to the overall SPIROMICS cohort, are shown in Table 1. Compared to the entire SPIROMICS cohort, participants who underwent bronchoscopy were younger, had milder disease, and were less likely to have had a COPD exacerbation during study follow-up (Table 1). BALF ferritin and iron were measured in 195 out of the total 215 recruited participants in the bronchoscopy sub-study. Plasma ferritin was available for 119 of these 195 participants in the bronchoscopy sub-study, as well as for 1575 participants in the entire SPIROMICS cohort.

**Table 1:** Participants enrolled in SPIROMICS and in the SPIROMICS bronchoscopy sub-study.

	SPIROMICS Cohort (n = 2974)	Bronchoscopy Substudy (n = 195)	P-value*
Age (y) *median [IQR]*	64 [56–70]	59 [52–67]	<0.001
Sex *N (%)*			0.89
Male	1577 (53.0%)	102 (52.3%)	
Smoking Status at baseline *N (%)*			0.91
Current Non-Smoker	1839 (62.7%)	121 (63.4%)	
Current Smoker	1093 (37.3%)	70 (36.6%)	
GOLD Stage *N (%)*			<0.001
0	924 (31.1%)	86 (44.1%)	
1	404 (13.6%)	32 (16.4%)	
2	820 (27.6%)	44 (22.6%)	
3	433 (14.6%)	8 (4.10%)	
4	186 (6.3%)	0 (0%)	
No COPD	207 (7.0%)	25 (12.8%)	
Haemoglobin (g/dL) *median [IQR]*	14.3 [13.2–15.3]	14.1 [13.2–14.9]	0.10
Exacerbations during follow up *N (%)*	969 (33.7%)	39 (20.3%)	<0.001

*Kruskal-Wallis or Chi-square test comparing patients included in the Bronchoscopy substudy to remaining SPIROMICS patients. IQR: interquartile range.

### BALF iron parameters in SPIROMICS

**Figure 1:**
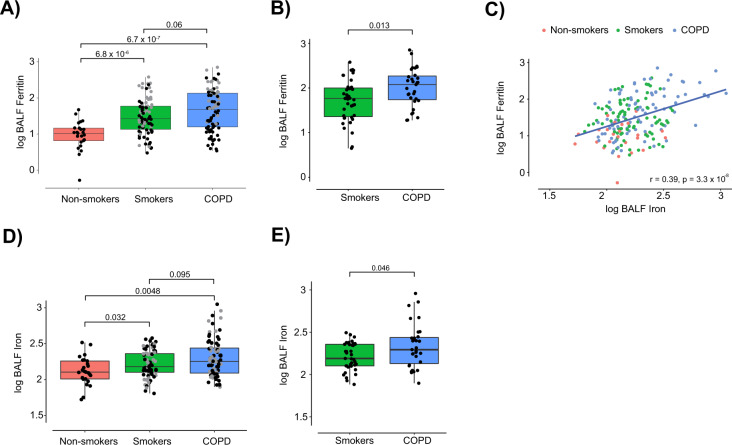
Bronchoalveolar lavage fluid (BALF) ferritin and iron levels are increased in smokers and participants with COPD. (**A**–**E**) Ferritin (ng/mL) and iron (mg/L) levels were measured in the BALF of SPIROMICS participants [never-smokers (*n* = 25), ever-smokers (including current and former smokers) without COPD (*n* = 86) and ever-smokers with COPD (*n* = 84, and *n* = 83 for ferritin and iron respectively)]. (**A**,**D**) Grey dots indicate current smokers at the time of baseline visit. (**B**,**E**) BALF ferritin and iron levels in current smokers without COPD (*n* = 39) and with COPD (*n* = 31) in SPIROMICS. (**C**) BALF ferritin association with BALF iron in never-smokers (*n* = 25, red), ever-smokers without COPD (*n* = 86, green) and ever-smokers with COPD (*n* = 84, and *n* = 83 for ferritin and iron respectively, blue) in SPIROMICS. Data (**A**,**B**,**D**,**E**) are presented as median with box indicating upper and lower quartiles, whiskers indicating extrema, and with P values calculated by non-parametric Kruskal-Wallis test. Linear associations (**C**) were tested with Pearson’s correlation coefficient.

We first examined relationships between BALF iron parameters and COPD status. Relative to never-smokers (median 10.5 ng/mL), BALF ferritin levels were significantly increased in smokers without COPD (26.4 ng/mL; p < 0.0001) and in COPD participants (47.3 ng/mL; p < 0.0001) (Fig. 1A). Amongst current smokers, BALF ferritin was higher in participants with COPD than in those without COPD (median 119.4 vs. 58.2 ng/mL; p = 0.013) (Fig. 1B). To eliminate the possibility that increased BALF ferritin occurred as a result of alveolar epithelial damage and transepithelial protein leakage, a sensitivity analysis was performed by normalizing BALF ferritin to BALF total protein and revealed similar, if not stronger, associations with disease status relative to unnormalized levels (Supplemental Fig. 1A-B). Ferritin has a critical role in iron storage, both protecting the cell from the dangers of labile iron and making it available in times of increased demand^10^. In this study, BALF ferritin correlated (r = 0.34; p < 0.0001) with BALF iron, confirming the two are interrelated (Fig. 1C). Similar to ferritin, BALF total iron was also significantly increased in smokers without COPD (median 150.95 μg/L; p = 0.032) and in participants with COPD (179.1 μg/L; p = 0.0048) relative to never-smokers (107.9 μg/L; Fig. 1D). BALF iron in current smokers with COPD was also higher compared to those without COPD (196.9 vs. 154.7 μg/L; p = 0.046) (Fig. 1E).

Similar results were also observed in an independent validation cohort (n = 59 BALF samples, Supplemental Table 1). In that cohort, BALF ferritin was higher in smokers (median 57.9 ng/mL; p < 0.0001) and participants with COPD (77.6 ng/mL; p < 0.0001) compared to healthy non-smoker controls (5.44 ng/mL), and again higher in current smokers with COPD relative to those without (median 112.7 vs. 57.9 ng/mL; p = 0.045) (Supplemental Fig. 2A-B). BALF ferritin also correlated with BALF iron in this cohort (r = 0.43, p = 0.00064), and similarly BALF iron was higher in smokers (67.8 μg/L; p = 0.031) and participants with COPD (73.9 μg/L; p = 0.0011) compared to non-smokers (22.6 μg/L), but did not differ significantly between current smokers with and without COPD (p = 0.35) (Supplemental Fig. 2C–E). The above findings demonstrate that individuals with COPD have higher levels of ferritin and iron in the lavage fluid lining the lung, an observation that was strengthened by current smoking status.

### BALF iron parameters and systemic iron status

**Figure 2:**
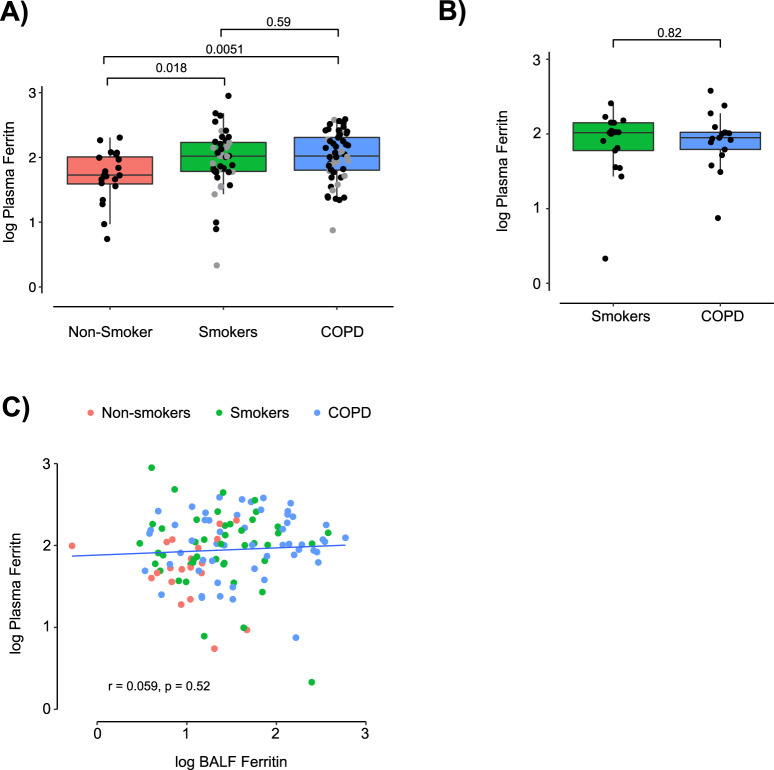
Plasma ferritin increases in smokers and in COPD but is not associated with BALF ferritin. (**A**) Plasma ferritin (ng/mL) in never-smokers (*n* = 20), ever-smokers without COPD (*n* = 44) and ever-smokers with COPD (*n* = 55) in the SPIROMICS bronchoscopy sub-study were measured using a Luminex-based multiplex assay system as described^62^. Grey dots indicate current smokers at the time of baseline visit. (**B**) Plasma ferritin in current smokers without (*n* = 17) and with COPD (*n* = 17) in the SPIROMICS bronchoscopy sub-study. (**C**) Association between plasma ferritin and BALF ferritin. (**A**,**B**) median, 25^th^ and 75^th^ percentiles, and extrema; P values by non-parametric Kruskal-Wallis test. Linear associations (**C**) were tested with Pearson’s correlation coefficient.

Circulating ferritin is widely used clinically as a surrogate of systemic and bone marrow iron stores, and is a component of routine anaemia work-up^28^. Additionally, ferritin is well-recognized as an acute phase reactant and is elevated in many chronic inflammatory diseases^29–31^. To evaluate whether increased BALF ferritin and iron were indicative of a global ferritin or iron increase, we next compared BALF and plasma ferritin levels in SPIROMICS bronchoscopy sub-study participants. As with BALF ferritin, plasma ferritin was significantly increased in smokers without COPD (median 104.5 ng/mL; p = 0.018) and in participants with COPD (105.0 ng/mL; p = 0.0051) relative to never-smokers (53.5 ng/mL) (Fig. 2A). However, unlike BALF ferritin, plasma ferritin was not higher in current smokers with COPD versus those without (104.0 vs. 89.0 ng/mL; p = 0.82) (Fig. 2B). Plasma ferritin did not associate with BALF ferritin, supporting the possible existence of two distinctive compartments for extracellular ferritin, and demonstrating that elevated BALF ferritin may not be merely a result of spillage from elevated plasma ferritin (Fig. 2C). The above plasma ferritin findings among bronchoscopy sub-study participants were also consistent with those of the entire SPIROMICS cohort (Supplemental Fig. 3A-B).

**Figure 3:**
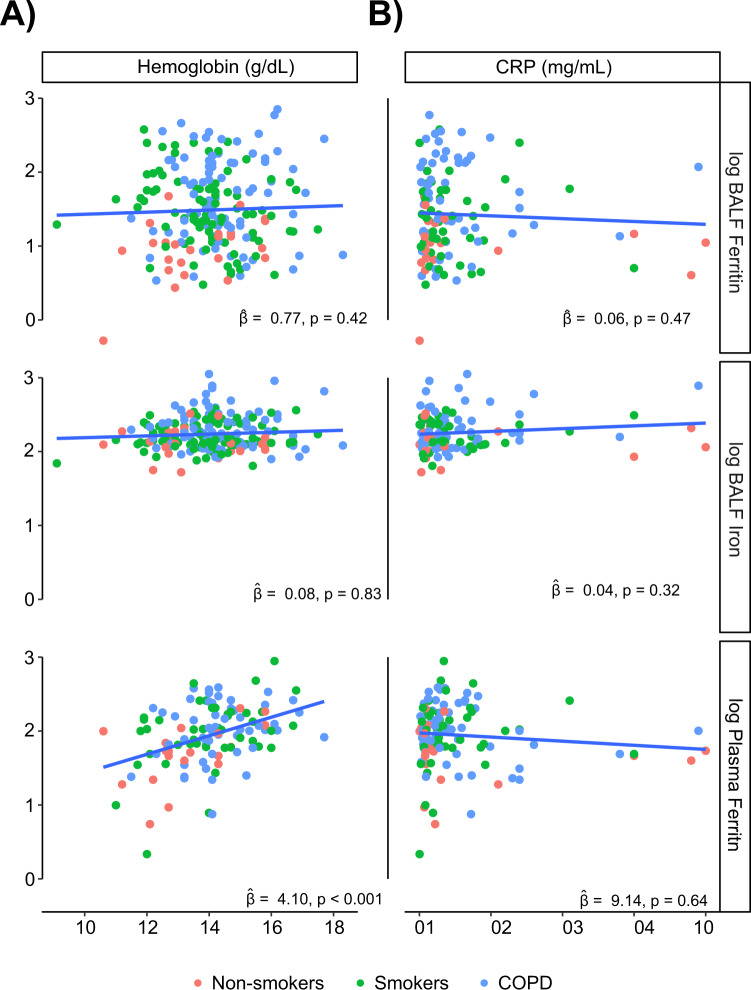
Local lung ferritin and iron levels do not correlate with systemic markers of iron storage or inflammation. (**A**) Haemoglobin (g/dL) and (**B**) CRP (μg/mL) levels in never-smokers (*n* = 25,20, red), ever-smokers without COPD (*n* = 85,44, blue) and ever-smokers with COPD (*n* = 83,55, green), as previously measured using a Luminex-based multiplex assay system^62^ and association with BALF ferritin (ng/mL), BALF iron (μg/L), and plasma ferritin (ng/mL) were tested with a linear model on the log-transformed markers and accounting for batch and site effects. $\hat{\beta }$ denotes adjusted increase in log-10 ferritin associated with unit increase in log-10 marker.

To test our hypothesis that increased BALF iron in COPD is indicative of a local pathogenic process independent of systemic iron levels, we investigated associations between BALF iron parameters and systemic iron (haemoglobin) and the systemic inflammatory marker C-reactive protein (CRP). BALF ferritin and iron did not associate with haemoglobin or CRP, whereas plasma ferritin strongly associated with haemoglobin and not CRP in bronchoscopy sub-study participants (Fig. 3A-B), and with both in the overall SPIROMICS cohort (Supplemental Fig. 4A-B).

### BALF Iron Parameters, COPD Progression, and Exacerbation Risk

**Figure 4:**
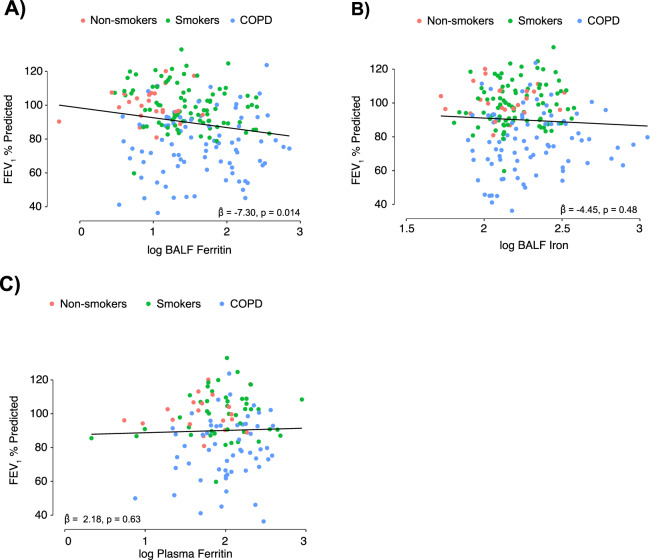
Higher BALF ferritin levels are associated with lower lung function. (**A**–**C**) Correlation between BALF ferritin (ng/mL), BALF iron (mg/L), and plasma ferritin (ng/mL) in never-smokers (*n* = 25 for BALF ferritin and iron, 20 for plasma ferritin, red), ever-smokers without COPD (*n* = 86 for BALF ferritin and iron, 44 for plasma ferritin green) and ever-smokers with COPD (*n* = 84 for BALF ferritin, 83 for BALF iron, 55 for plasma ferritin, blue) and post-bronchodilator FEV_1_% predicted. Linear associations (**A**–**C**) were tested, adjusting for age, sex, smoking status and study site. $\hat{\beta }$ denotes adjusted increase in log-10 ferritin associated with unit increase in log-10 marker.

Cigarette smoking and COPD are associated with an accelerated decline in lung function, most commonly measured by spirometry and represented as FEV_1_ % predicted (forced expiratory volume in one second)^32,33^. In the SPIROMICS cohort, BALF ferritin negatively correlated with FEV_1_ % predicted in models adjusted for age, sex, smoking status and clinical site (10-fold increase in BALF ferritin $\hat{\beta }$ = −7.3 points; confidence interval [CI] −13.10, −1.5; p = 0.01) (Fig. 4A). This was maintained upon removing never-smokers (adjusted $\hat{\beta }$ = −7.53 points; CI −14.11, −0.94; p = 0.02) (Supplemental Table 2). BALF iron did not significantly correlate with FEV_1_% predicted (adjusted $\hat{\beta }$ = −4.45 points; CI −16.87,7.97; p = 0.48) (Fig. 4B). Plasma ferritin also did not correlate with FEV_1_% predicted in adjusted models in the bronchoscopy sub-study ($\hat{\beta }$ = 2.18 points; CI −6.86,11.22; p = 0.63) (Fig. 4C) or in the overall SPIROMICS cohort (Supplemental Fig. 5). BALF ferritin, BALF iron or plasma ferritin were not associated with quantifiable radiographic measures of COPD such as small airway disease (PRM^FSAD^) or emphysema (PRM^EMPH^) (Supplemental Fig. 6A-B and Supplemental Table 2).

**Figure 5:**
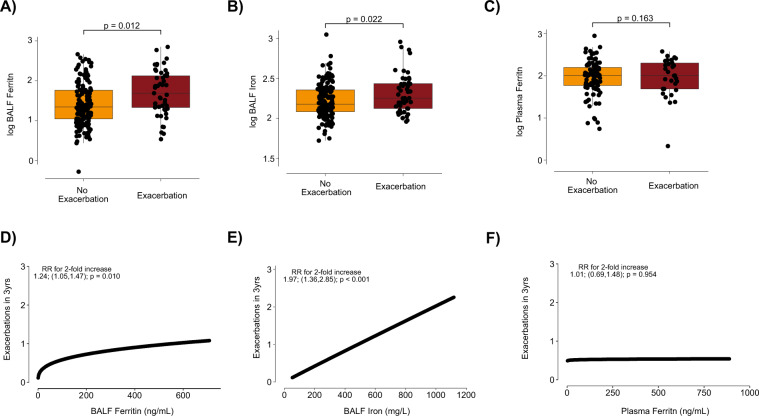
Higher BALF ferritin and iron levels are associated with increased exacerbation risk. (**A**,**B**) BALF ferritin (ng/mL) and BALF iron (µg/mL) in participants who had had one or more acute COPD exacerbation (*n* = 49 for ferritin, *n* = 48 for iron) when compared to participants who did not (*n* = 146). (**C**) Plasma ferritin levels in participants with (*n* = 30) versus without (*n* = 89) exacerbations. (**A**–**C**) median, 25^th^ and 75^th^ percentiles, extrema; Adjusted P values (age, sex, smoking status and site) (**D**–**F**) Predicted exacerbation rate per SPIROMICS bronchoscopy sub-study participant over 3–5 years of follow up by BALF ferritin (ng/mL) (**D**), BALF iron (µg/mL) (**E**) or plasma ferritin (ng/mL) (**F**) were estimated with a zero-inflated negative binomial model for a participant with a median FEV_1_ % predicted for (**D**) BALF ferritin (*n* = 195), (**E**) BALF iron (*n* = 194), and (**F**) plasma ferritin (*n* = 119).

FEV_1_ is closely linked to COPD exacerbations. Patients with frequent exacerbations have steeper decline in FEV_1_, especially in those with early COPD, where each individual exacerbation results in a profound loss of lung function^34–36^. In SPIROMICS, participants in the bronchoscopy sub-study had less severe disease compared to the overall cohort, and therefore had fewer exacerbations (Table 1). Nevertheless, compared to participants without exacerbations during follow-up, those who did have exacerbations had higher levels of BALF ferritin in adjusted models (2-fold increase in ferritin $\hat{\beta }\,$= 1.39 points; CI 1.08, 1.80; p = 0.012). Similarly, compared to participants without exacerbations during follow-up, those who had exacerbations had higher levels of BALF iron (adjusted $\hat{\beta }\,$= 1.90 points; CI 1.11, 3.36; p = 0.022). Plasma ferritin did not associate with exacerbation events in adjusted models ($\hat{\beta }\,$= 0.95 points; CI 0.88, 1.02; p = 0.163) (Fig. 5A–C).

When analysed relative to the baseline visit in a zero-inflated negative binomial model, a 2-fold increase in BALF ferritin was associated with a 24% increase in the rate of yearly exacerbations (rate ratio [RR] 1.24; CI 1.05, 1.47; p = 0.010) (Fig. 5D, Supplemental Table 3). Comparable results were observed adjusting for participant age, sex and smoking status, with the point estimate not substantially changed (RR 1.23; CI 0.99, 1.53; p = 0.058) (Supplemental Table 3). Similar results were found for BALF iron, for which a 2-fold increase was associated with a 2-fold increase in the yearly rate of exacerbations (RR 1.97; CI 1.36, 2.85; p < 0.001 and adjusted RR 1.98; CI 1.33, 2.93; p < 0.001) (Fig. 5E, Supplemental Table 3). Notably, plasma ferritin was not associated with exacerbation rate in the bronchoscopy sub-study or in the overall cohort (Fig. 5F, Supplemental Table 3). After analysing only exacerbations following the bronchoscopy visit, many exacerbation events were lost and the association between exacerbation rate and BALF ferritin was no longer significant (adjusted RR 1.11; CI 0.88–1.40; p = 0.401), but a significant association was maintained for BALF iron (adjusted RR 1.81; CI 1.18–2.76; p = 0.006) (Supplemental Table 3).

## Discussion

In this study, we found that airway iron parameters were significantly higher in the bronchoalveolar lavage fluid of individuals with COPD when compared to “healthy” smokers and controls, and that higher levels of BALF ferritin and iron associate with a greater risk for COPD exacerbation. These results, contrasting with the absence of similar associations with plasma ferritin, support the hypothesis that changes in “local” lung iron homeostasis contribute to COPD pathobiology.

The regulation of iron acquisition, utilization, and storage is fundamental to the correct function of lung epithelial, endothelial, and immune cells^37^. Iron is also crucial for the metabolism and growth of native and pathogenic microbes, including those associated with recurrent infections in COPD such as *Haemophilus influenzae* and *Pseudomonas aeruginosa*^38,39^. During infection, the host deploys a number of defence mechanisms in an attempt to sequester free iron away from bacteria, including promoting iron uptake, increasing intracellular iron storage capacity, and secreting proteins (e.g. ferritin, lactoferrin, lipocalin-2) to chelate remaining extracellular iron^40–42^. Saturation of these adaptive mechanisms directly (e.g. smoke) or indirectly (genetic susceptibility) may result in an excess of iron in the lung microenvironment, which in turn may influence the size and diversity of microbial populations that colonize and proliferate there. In support of this, we have also observed increased BALF levels of the siderophore lipocalin-2 in smokers and participants with COPD, when compared to control subjects in the SPIROMICS cohort (*data not shown*). Given the fact that infections are the dominant cause of COPD exacerbations, it follows that disrupting such a complex iron regulatory system and changing iron availability could potentially directly affect exacerbation risk in COPD patients. This is highlighted by the strong association between BALF iron levels and exacerbation frequency found in this study, without a concurrent association with FEV_1_, suggesting that this iron-mediated process may precede lung function decline in these patients.

In this study, the source of increased BALF iron and ferritin is unknown. Alveolar macrophages are iron- and ferritin-rich in smokers and COPD patients, but both lung epithelial and endothelial cells are also potential sources. As previously mentioned, mainstream cigarette smoke contains little iron^24,25^. We hypothesize that iron accumulates as a biological response to continuous smoke exposure. Specifically, smoke exposure leads to altered responses to hypoxia, higher erythropoietin (EPO) levels^43^ and expanded, but inefficient erythropoiesis in the bone marrow (*unpublished data*)^44^. This may in turn lead to changes in iron uptake and release in macrophages globally, including in the lung, to increase iron availability for hemoglobin synthesis^45^. Alternatively, alveolar macrophage iron metabolism is directly dysregulated upon smoke exposure. Alveolar macrophages from COPD patients have markedly depressed M1-responses^46^ and markers^47,48^ and lower phagocytic ability^49^. Such deactivation in the M1-polarization programme is accompanied by an increase in the expression of wound-healing M2 markers^47,48^. Iron regulatory responses also differ between macrophage phenotypes where M1-macrophages accumulate iron as part of a bacteriostatic stratagem, whilst the opposite is the case for M2-macrophages, in which iron release is favoured^50^. Changes in alveolar macrophage biology therefore may favor a phenotype of active iron release by the macrophage, leading to increased extracellular iron. Similarly, we believe that the increased level of BALF ferritin detected in this and other studies is a result of a biological response to smoke exposure. In the lung, intracellular ferritin is comprised of light and heavy chain subunits (FTL and FTH, respectively) in equal proportions, and has relatively low iron saturation compared to ferritin from other organs such as the liver or spleen^51^. Extracellular ferritin is also a combination of FTL and FTH, which in serum consists predominantly of FTL^52,53^. FTL may be more abundant than FTH in the BALF of healthy subjects^54^; however, we did not evaluate proportions of FTL and FTH in ferritin found in BALF, and most commercial ELISA kits, including those used in this study, are unable to discriminate between these subunits. The purpose of ferritin and iron release into the alveolar space is unclear and is currently the subject of further investigation. Ferritin can serve as an iron carrier; macrophages, in particular, use ferritin to deliver iron to hepatocytes and erythroid progenitors^55,56^.

Our findings, in particular the discordance between BALF and plasma ferritin with clinical associations, extend on previous studies of serum ferritin levels in COPD patients. Serum ferritin is increased in smokers, and while some studies showed higher serum ferritin correlated with worse airflow obstruction, in others higher serum ferritin and iron correlated with spirometric protection against cigarette smoke^57–59^. Such findings do not inherently contradict our results, as blood and airway ferritin may be indicative of distinct pathologic mechanisms, and the phenomenon of local iron overload we demonstrated does not exclude systemic iron deficiency in some COPD patients. Distinguishing between local and systemic iron status is a crucial subject for future investigation, as treating one anatomic compartment with iron chelation or supplementation may exacerbate disease processes in another.

Our study has several important strengths. This is the largest study of iron metabolism in a prospective COPD cohort and exploits the fact that SPIROMICS is enriched for spirometrically mild-to-moderate disease, a deliberate design feature to discover factors that lead to disease progression. We included an independent validation cohort to corroborate some of our findings. To eliminate the possibility that increased BALF ferritin occurred as a result of alveolar epithelial damage and transepithelial protein leakage, we normalized BALF ferritin to BALF total protein, which strengthened BALF ferritin correlations with disease status.

Our study also has limitations. We determined smoking status by self-report, unverified in this analysis using objective measurements such as urine cotinine; we also limited current smoking status to the baseline visit, recognizing that study participants could have quit smoking between that visit and their bronchoscopy. Subjects who agreed to the bronchoscopy sub-study were self-selected and may not be representative of the general COPD population. Critically, the bronchoscopy sub-study population is overly represented by never-smokers and ever-smokers with preserved lung function and is constrained further by a safety check on adequate FEV_1_ on the date of bronchoscopy. This stringent selective process limits our statistical power, given the limited numbers of severe COPD cases and exacerbations. The timing of bronchoscopy, in some cases up to 1 year after the initial visit, further curtails the exacerbation event numbers. In addition, we measured BALF ferritin and iron at a single time point, and it is not known whether these levels are stable over time in a given patient, or fluctuate, especially around the time of an exacerbation or infection. We correlated BALF ferritin with many clinical variables and cannot exclude the possibility that iron metabolism is merely an intermediary between these variables and other more established predictors such as FEV_1_% predicted. Furthermore, our models assume equal follow-up time for study participants, whereas in reality that follow-up time varied substantially. Despite these limitations, we consider the over-representation of never-smokers and ever-smokers with preserved lung function a strength, in that this allowed us to test our hypothesis at an early disease stage, before significant lung function loss has taken place. Our study is also the first to associate BALF iron parameters with important COPD outcomes such as exacerbation risk, associations that do not exist with plasma ferritin. The lack of associations with radiographic measures of small airways disease and emphysema further support our hypothesis that higher iron may be associated with early disease and may be a factor in COPD pathogenesis. Given the impracticality of routine bronchoscopy outside the research setting, our goal is not to advocate for BALF ferritin or iron levels as COPD biomarkers, nor to suggest predictive or causal relationships. Instead, we highlight these associations to provide evidence for a role for abnormal airway iron metabolism in COPD pathogenesis and progression, to support exploration of the basic mechanisms behind this phenomenon as well as to draw attention to the potential of targeting lung iron overload in COPD.

In conclusion, BALF ferritin and iron levels are higher in subjects with COPD, an observation that correlates with heightened COPD exacerbation susceptibility. If replicated, these results suggest that iron overload might represent an under-recognized disease endotype. The pathobiologic mechanism behind these associations warrants further investigation.

## Methods

### Ethics statement

All clinical investigations are conducted according to the principles of the Declaration of Helsinki. The individual institutional review boards (IRBs) of all participating clinical centres approved all study protocols. All participants understood the purpose of the study and provided written informed consent before they underwent any research activities or procedures.

### Study design and sample collection

SPIROMICS (ClinicalTrials.gov NCT01969344T4) is an ongoing longitudinal multicenter observational study funded by the National Heart, Lung, and Blood Institute^27^ that recruited 2981 subjects, 40 to 80 years of age, including some who had never smoked cigarettes (≤1 pack-year of tobacco-smoking history), current or former smokers (ever-smokers, greater than 20 pack-years) without airflow obstruction, and ever-smokers with airflow obstruction^60^. Data was collected at the initial study visit, including demographics, comorbidities, questionnaires, current cigarette smoke exposure and 6-minute walk distance (6MWD)^27^. The extent of airway-wall abnormality was characterized with HRCT scans of the lung, using Imbio (parametric response mapping, PRM) and VIDA Diagnostics software; small airway disease (PRM^FSAD^) was defined as areas of lung that are greater than −950 HU on inspiration but less than −856 HU on expiration, denoting air-trapping, while emphysema was defined as areas of lung that less than −950 HU on inspiration and less than −856 HU on expiration^61^.

Clinical data was collected at the baseline study visit and in follow-up visits as previously described^27^. Peripheral blood was collected as part of the baseline visit, and plasma biomarkers were measured using a Luminex-based multiplex assay; relevant to this study, plasma ferritin was shown to be equivalent to serum ferritin using this assay method^62^. Exacerbations were defined as health care utilization events (office visit, hospital admission, or emergency department visit for a respiratory flare-up) that were treated with antibiotics, systemic corticosteroids, or both. Exacerbation history was prospectively collected every 3 months for up to 5 years using a structured questionnaire^2,60^.

A subgroup of subjects (n = 215) with post-bronchodilator FEV_1_ > 30% predicted and without an exacerbation in the prior six weeks were further enrolled in the bronchoscopy sub-study, in which on the first of two visits, sputum induction was performed as previously described^63^. On the second visit, post-bronchodilator FEV_1_ was measured and only subjects with an FEV_1_ > 30% predicted were allowed to participate in the bronchoscopy portion of the study. BAL was performed in the right middle lobe and lingula by instilling two aliquots of 40 mL and one aliquot of 50 mL per lobe (260 mL total volume), after excluding an initial airway wash sample. Unfiltered BALF fluid was collected into a sterilized beaker or in multiple 50 mL conical tubes on ice, then centrifuged at 300 x g for 5 min and the supernatant aliquoted into 1 mL aliquots for storage at −80 °C, representing one BALF sample per patient as described previously^63,64^. Because no research plasma samples were obtained in the bronchoscopy sub-study, the plasma biomarkers, including ferritin, in this analysis were measured from the baseline visit samples. For participants in the bronchoscopy sub-study, exacerbation events were analysed both relative to the baseline visit and to the bronchoscopy visit (0-14 months after baseline visit).

To replicate the study findings in an independent cohort, never smokers (n = 20), healthy smokers (n = 21) with normal lung function and individuals with COPD (n = 18), recruited by the Department of Genetic Medicine, Weill Cornell Medical College underwent bronchoscopy with BALF isolated as described above (*see* Supplementary Information and Supplemental Table 1).

### Ferritin measurement and normalization

BALF ferritin was quantified by ELISA using the Abcam Human Ferritin ELISA Kit (Cat#ab200018), which detects both ferritin heavy and light chain. BALF ferritin was normalized to total protein, measured using the Thermo Scientific Pierce BCA Protein Assay Kit (Cat#23225).

### Total iron measurements

After centrifugation (1000 × *g* for 5 mins), 60 μL of BALF was digested with 40 μL of 50% Nitric Acid (in distilled H_2_O) containing a final concentration of 0.1% digitonin for 2 hours at 60 °C. Total iron, including both bound and unbound forms, was measured in triplicate in 20 μL of digested fractions using a graphite furnace atomic absorption spectrophotometer (GFAAS, Perkin Elmer PinAAcle 900z), comparing unknown values to a standard curve of known concentrations of iron (1000 PPM in 2% Nitric Acid).

### Statistical analysis

Clinical characteristics of SPIROMICS participants enrolled in the bronchoscopy sub-study were compared to those of all SPIROMICS participants, summarized using means and standard deviations or counts and percentages as appropriate. BALF Ferritin and iron were analysed on the log 10 scale. Plasma ferritin, haemoglobin and CRP were measured as previously described^62^, and analysed on the log scale, accounting for site and batch effects. Associations between BALF ferritin and baseline characteristics were performed using Kruskal-Wallis tests for categorical variables, and Pearson correlations for continuous variables, and were unadjusted unless otherwise specified. Sensitivity analyses including all SPIROMICS participants were also performed to study associations in the overall cohort. COPD exacerbations were analyzed in three ways. First, participants were dichotomized into those with any exacerbations between their baseline visit and the end of study follow-up versus those without exacerbations in this timeframe, and ferritin levels were compared across groups. Second, participants were dichotomized into those with any exacerbations between the bronchoscopy visit and the end of the study follow-up, versus those without, and ferritin levels were compared across groups. Third, the rate of exacerbations per participant per year was estimated using a negative binomial zero-inflated model, with % FEV_1_ predicted as the predictor in the binomial model. Ferritin associations were studied in models unadjusted as well as adjusted for age, sex, and smoking status at baseline or at the time of the bronchoscopy, as appropriate. Adjustment for study site prevented model convergence and was thus removed. Yearly exacerbation rate ratios, as well as 95% confidence intervals (CI) were estimated, and the unadjusted model was then used to plot predicted exacerbation rates in three years in a participant with a median FEV_1%_ predicted. BALF ferritin levels in a validation cohort (*see* Supplemental Table 1) were similarly analysed on a log 10 scale, and compared across non-smokers, smokers without COPD, and participants with COPD.

## Supplementary information

Supplementary information.
