# Supplementary material for: Increased airway iron parameters and risk for exacerbation in COPD: an analysis from SPIROMICS
Source: Sci Rep. 2020 Jun 29;10:10562. doi: 10.1038/s41598-020-67047-w (PMC7324559; doi:10.1038/s41598-020-67047-w)
Supplement: Supplementary file 1 — Supplementary information. [file 41598_2020_67047_MOESM1_ESM.pdf]

Supplemental Information

TITLE: Increased airway iron parameters and risk for exacerbation in COPD: an analysis from SPIROMICS

AUTHORS: William Z. Zhang[1,2], Clara Oromendia[1,3], Sarah Ann Kikkers[1], James J. Butler[1], Sarah O'Beirne[1,4], Kihwan Kim[1], Wanda K. O'Neal[5], Christine M. Freeman[6, 7], Stephanie A. Christenson[8], Stephen P. Peters[9], J. Michael Wells[10], Claire Doerschuk[5], Nirupama Putcha[11], Igor Barjaktarevic[12], Prescott G. Woodruff[8], Christopher B. Cooper[12], Russell P. Bowler[13, 14], Alejandro P. Comellas[15], Gerard J. Criner[16], Robert Paine III[17], Nadia N. Hansel[11], Meilan K. Han[6], Ronald G. Crystal[4], Robert J. Kaner[1,4], Karla V. Ballman[1,3], Jeffrey L. Curtis[6, 7], Fernando J. Martinez[1,2] and Suzanne M. Cloonan[1,18] for the SPIROMICS investigators*.

[1]Division of Pulmonary and Critical Care Medicine, Joan and Sanford I. Weill Department of Medicine, [2]New York-Presbyterian Hospital, Weill Cornell Medical College, New York, New York; [3]Department of Healthcare Policy and Research, Division of Biostatistics and Epidemiology, Weill Cornell Medicine, New York, New York. [4]Department of Genetic Medicine, Weill Cornell Medical College, New York, New York; [5]University of North Carolina Marsico Lung Institute, Chapel Hill, North Carolina; [6]Pulmonary and Critical Care Medicine Division, Department of Internal Medicine, University of Michigan Health System, Ann Arbor, Michigan; [7]Veterans Affairs Ann Arbor Healthcare System, Ann Arbor, Michigan; [8]University of California at San Francisco, San Francisco, California; [9]Wake Forest School of Medicine, Winston-Salem, North Carolina; [10]Division of Pulmonary and Critical Care, University of Alabama at Birmingham, Birmingham, Alabama; [11]Johns Hopkins University School of Medicine, Baltimore, Maryland; [12]Division of Pulmonary and Critical Care Medicine, University of California Los Angeles Medical Center, Los Angeles, California; [13]University of Colorado School of Medicine, Aurora, Colorado; [14]National Jewish Health, Denver, Colorado; [15]Division of Pulmonary and Critical Care,

University of Iowa, Iowa City, Iowa; [16]Department of Pulmonary & Critical Care Medicine, Temple University, Philadelphia, Pennsylvania; [17]Section of Pulmonary and Critical Care Medicine, Salt Lake City Department of Veterans Affairs Medical Center, Salt Lake City, Utah; [18] School of Medicine, Trinity Biomedical Sciences Institute and Tallaght University Hospital, Trinity College Dublin Ireland.

[*]Author for correspondence: Suzanne M. Cloonan, szc2009@med.cornell.edu; 1300 York Ave, New York, NY 10065; Tel: +1 212-746-4265; Fax: +1 212-746-7293.

Methods

Study Design and Sample Collection

Baseline characteristics included clinical (age, sex, smoking status, GOLD stage), subject-reported outcomes (CAT, SGRQ), systemic iron storage markers (hemoglobin), and the inflammatory marker CRP.

Replication/Validation Cohort

All clinical investigations were conducted according to the principles of the Declaration of Helsinki. The individual institutional review boards (IRBs) of Weill Cornell Medical College approved all study protocols. All participants understood the purpose of the study and provided written informed consent before they underwent any research activities or procedures. To replicate the study findings in an independent cohort, never smokers (*n*=20), healthy smokers (*n*=21) with normal lung function and individuals with COPD (*n*=18) were recruited by the Department of Genetic Medicine, Weill Cornell Medical College (for inclusion and exclusion criteria see [1]) and underwent bronchoscopy with bronchoalveolar lavage (BAL) (see **Supplemental Table 1** for complete demographic information). BAL was performed by instilling 30 mL aliquots of sterile 0.9% saline into the right middle lobe and/or lingula, to a total volume of 150 mL in the case of current smokers or 300 mL in non-smokers. After collection, BALF was filtered through a double layer of gauze and centrifuged at 1200 rpm for 5 min at 4°C. The supernatant was then removed, protease inhibitor cocktail (Sigma-Aldrich, catalog number S8830) added and stored in aliquots at -80°C until use. EDTA blood samples were collected within 30 days of bronchoscopy, with samples collected on the procedure day in 27% of subjects.

Supplemental Figure Legends

Supplemental Figure 1. BALF ferritin normalization. (A-B) BALF ferritin (ng/mL) and total protein (mg/mL) were measured in non-smokers (*n*=25), smokers (*n*=86) and COPD patients (*n*=31), and BALF ferritin was normalized to total protein in participants enrolled in the SPIROMICS bronchoscopy sub-study. Data are presented as median with box indicating 25th and 75th percentiles, whiskers indicating extrema, and with P values calculated by non-parametric Kruskal-Wallis test.

Supplemental Figure 2. BALF iron parameters in an independent validation cohort. (A-E) BALF ferritin (ng/mL) and iron (mg/L) measured in an independent Weill Cornell cohort [never-smokers (*n*=20), ever-smokers with normal lung function (*n*=21) and ever-smokers with COPD (*n*=18)]. (B, E) BALF ferritin and iron was measured in current smokers with (*n*=20) and without COPD (*n*=20). (C) In this independent validation cohort, BALF ferritin strongly correlated with BALF iron levels (*n*=59). Data (A-B, D-E) are presented as median with box indicating 25th and 75th percentiles, whiskers indicating extrema, and with P values calculated by non-parametric Kruskal-Wallis test. Linear associations (C) were tested with Pearson's correlation coefficient.

Supplemental Figure 3. Plasma ferritin levels in the overall SPIROMICS cohort coincided with results from the bronchoscopy sub-study. (A) Plasma ferritin levels in the overall SPIROMICS cohort were previously measured using a Luminex-based multiplex assay system [2] in non-smokers (*n*=143, red), smokers (*n*=495, green) and COPD patients (*n*=1056, blue). (B) Plasma ferritin levels in the overall SPIROMICS cohort in smokers without (*n*=234) and with COPD (*n*=341). Data (A-B) are presented as median with box indicating 25th and 75th percentiles, whiskers indicating extrema, and with P values calculated by non-parametric Kruskal-Wallis test.

Supplemental Figure 4. Plasma ferritin levels in the overall SPIROMICS cohort coincided with results from the bronchoscopy sub-study. (A) Hemoglobin (g/dL) and (B) CRP (μg/mL) levels in non-smokers (*n*=143/143, red), smokers (*n*=490/495, green) and COPD patients (*n*=1049/1056, blue) previously measured using a Luminex-based multiplex assay system were tested with a linear model on the log-transformed markers and accounting for batch and site effects. *B* denotes adjusted increase in log-10 ferritin associated with unit increase in log-10 marker.

Supplemental Figure 5. Plasma ferritin is not associated with lung function.
Plasma ferritin and association with post-bronchodilator FEV_1 in non-smokers (*n*=143, red), smokers (*n*=495, green) and COPD patients (*n*=1056, blue). Linear association was tested, adjusting for age, sex, smoking status and study site.

Supplemental Figure 6. Higher BALF ferritin levels are associated with lower lung function. (A-B) Correlation between BALF ferritin (ng/mL), BALF iron (mg/L), and plasma ferritin (ng/mL) in never-smokers (*n*=25 for BALF ferritin and iron, 20 for plasma ferritin, red), ever-smokers without COPD (*n*=86 for BALF ferritin and iron, 44 for plasma ferritin green) and ever-smokers with COPD (*n*=84 for BALF ferritin, 83 for BALF iron, 55 for plasma ferritin, blue) and functional small airway disease or emphysema by parametric response mapping (PRM^{FSAD} and PRM^{EMPH}, respectively). Linear associations (A-B) were tested, adjusting for age, sex, smoking status and study site. $\hat{\beta}$ denotes adjusted increase in log-10 ferritin/iron associated with unit increase in log-10 marker.

Supplemental References

1 Yang, J. *et al.* Smoking-Dependent Distal-to-Proximal Repatterning of the Adult Human Small Airway Epithelium. *American journal of respiratory and critical care medicine* **196**, 340-352, doi:10.1164/rccm.201608-1672OC (2017).

2 O'Neal, W. K. *et al.* Comparison of serum, EDTA plasma and P100 plasma for luminex-based biomarker multiplex assays in patients with chronic obstructive pulmonary disease in the SPIROMICS study. *Journal of translational medicine* **12**, 9, doi:10.1186/1479-5876-12-9 (2014).

Supplemental Table 1. Patients enrolled in the Weill Cornell Medical College Validation Cohort

	**Validation Cohort (n = 59)**
Age (y) *median [25th-75th percentile]*	50 [41 - 55]
Sex *N (%)*	
Male	40 (67.8%)
Smoking Status at baseline *N (%)*	
Current Non-Smoker	24 (40.7%)
Current Smoker	35 (59.3%)
GOLD Stage *N (%)*	
1	12 (20.3%)
2	2 (3.4%)
3	4 (6.8%)
No COPD	41 (59.5%)

Supplemental Table 2. Iron parameters and clinical characteristics within subgroups

	**ALL Groups** *Adjusted for age, sex, smoking status and clinical site*		**Smokers + COPD Groups** *Adjusted for age, sex, smoking status and clinical site*	
	$\hat{\beta}$ (CI)	*P-value**	$\hat{\beta}$ (CI)	*P-value**
FEV1 % Predicted				
BALF Ferritin	-7.30 (-13.10, -1.50)	0.01	-7.53 (-14.11, -0.94)	0.02
BALF Iron	-4.45 (-16.87,7.97)	0.48	-5.28 (-19.41,8.85)	0.46
Plasma Ferritin	2.18 (-6.86,11.22)	0.63	4.45 (-6.60,15.51)	0.42
Radiographic small airway disease (PRM^{FSAD})				
BALF Ferritin	0.97 (-2.70,4.64)	0.60	-0.48 (-4.65,3.69)	0.82
BALF Iron	2.31 (-5.44,10.06)	0.55	1.86 (-6.80,10.52)	0.67
Plasma Ferritin	-2.69 (-8.30,2.91)	0.34	-3.86 (-10.57,2.85)	0.25
Radiographic Emphysema (PRM^{EMPH})				
BALF Ferritin	1.13 (-0.17,2.43)	0.09	-0.04 (-0.42,0.35)	0.84
BALF Iron	1.78 (-0.98,4.54)	0.20	-0.06 (-0.93,0.81)	0.89
Plasma Ferritin	-0.79 (-2.35,0.77)	0.31	-0.45 (-1.02,0.11)	0.11

*Pairwise contrasts of strata using Tukey's range test (unadjusted and adjusted)
Abbreviations: BALF; Bronchoalveolar Lavage Fluid; $\hat{\beta}$=estimated change log10 ferritin or iron with a one unit increase in clinical characteristic, CI=95% confidence interval

Supplemental Table 3. Zero inflated negative binomial models for Iron markers in BALF and plasma.

	Unadjusted		Adjusted*	
	Rate Ratio (CI)	*P-value*	Rate Ratio (CI)	*P-value*
BALF Ferritin+	1.24 (1.05,1.47)	*0.01*	1.23 (0.99,1.53)	*0.06*
BALF Ferritin+ (After bronchoscopy only)	1.13 (0.96,1.35)	*0.15*	1.11 (0.88,1.40)	*0.40*
BALF Iron+	1.97 (1.36, 2.85)	*<0.001*	1.98 (1.33,3.93)	*<0.001*
BALF Iron+ (After bronchoscopy only)	1.77 (1.22, 2.57)	*0.003*	1.81 (1.18,2.76)	*0.006*
Plasma Ferritin+ Bronchoscopy sub-study	1.01 (0.69, 1.48)	*0.95*	1.21 (0.81,1.83)	*0.35*
Plasma Ferritin+ (After bronchoscopy only)	0.85 (0.53, 1.35)	*0.49*	0.91 (0.55,1.50)	*0.71*
Plasma Ferritin+ Overall cohort	0.96 (0.91, 1.02)	*0.21*	1.00 (0.94, 1.06)	*0.98*
Plasma Ferritin Overall Cohort + (After bronchoscopy only)	0.83 (0.52, 1.32)	*0.42*	0.89 (0.54, 1.46)	*0.65*

+2-fold increase; *Adjusted models using smoking status, sex, age as coefficients; CI: 95% confidence interval

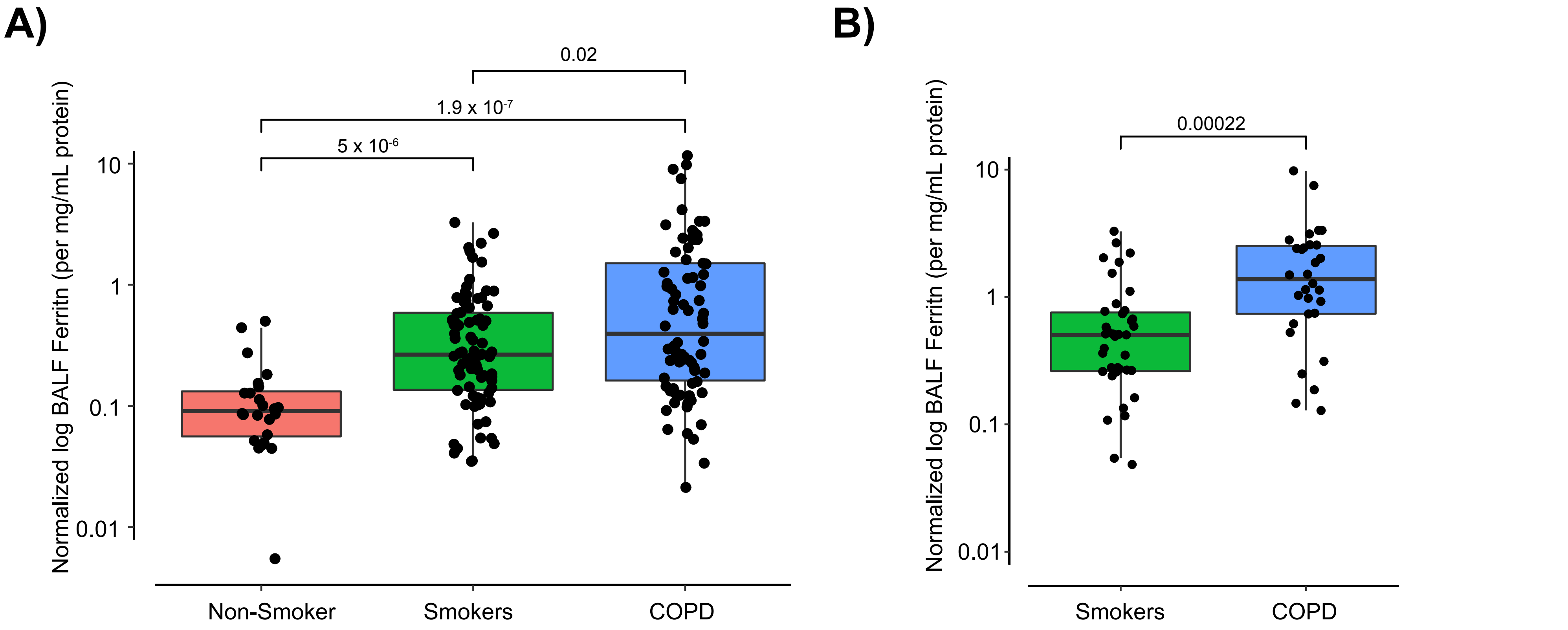

Supplemental Figure 1.

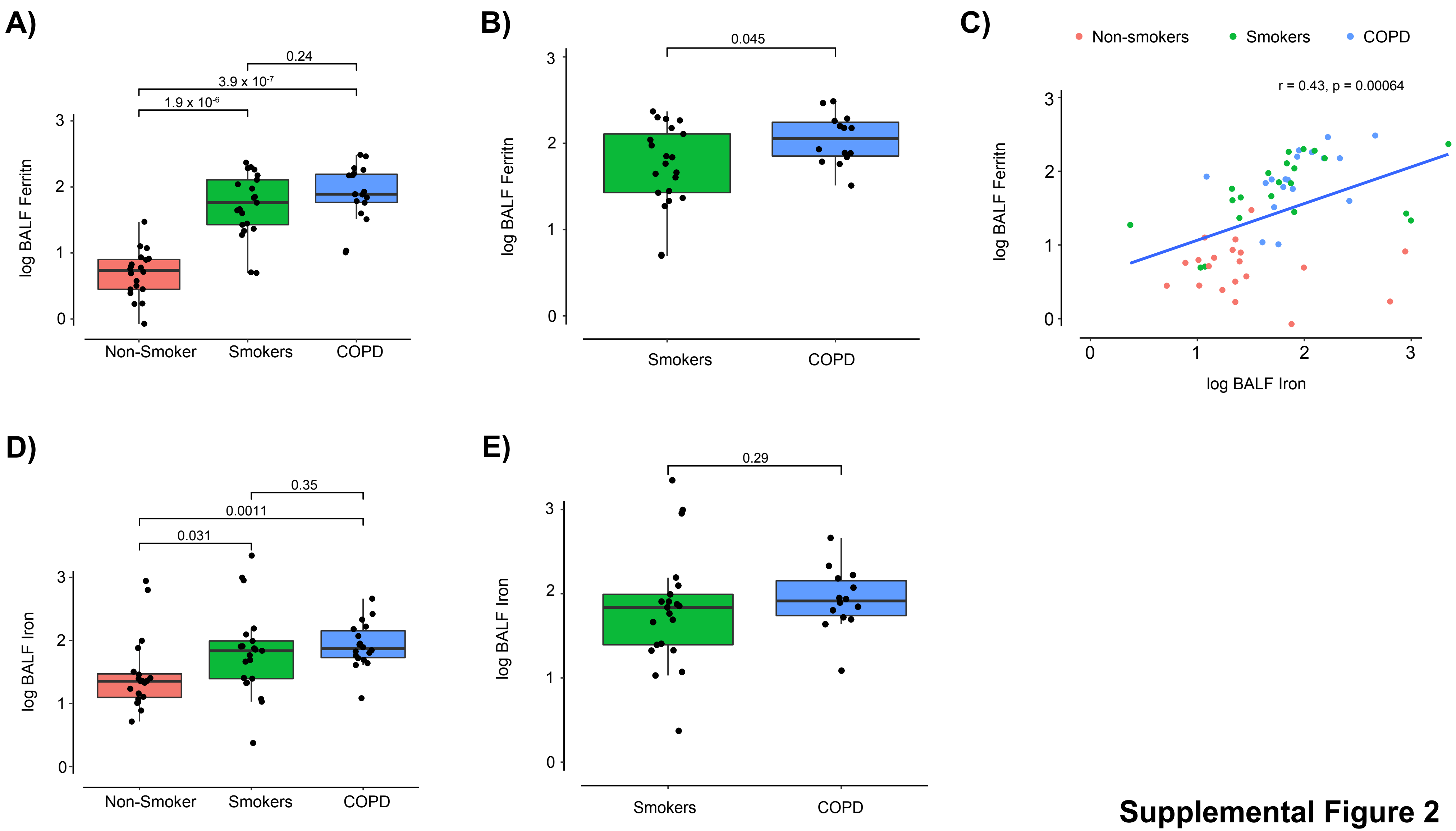

Supplemental Figure 2

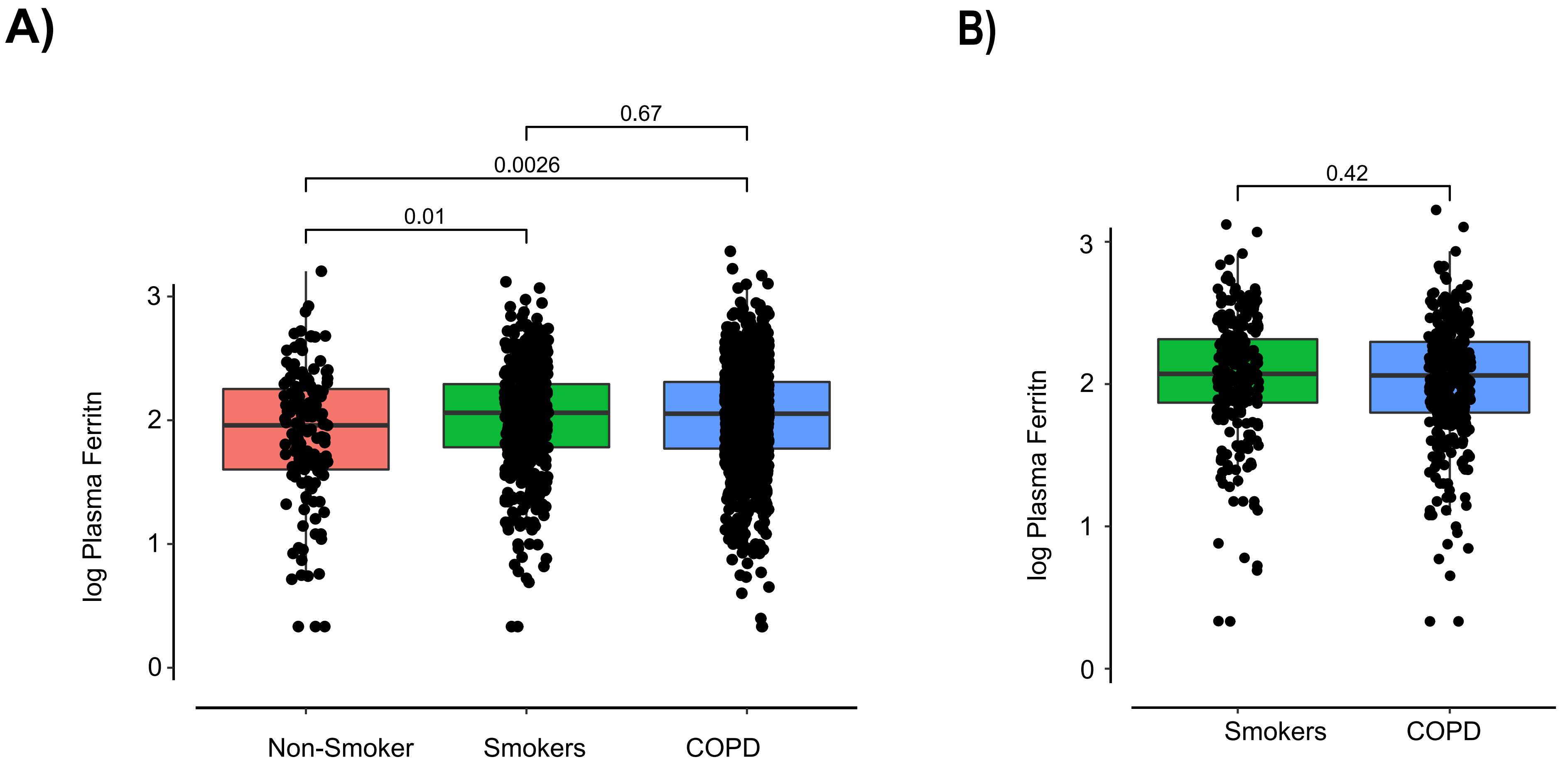

Supplemental Figure 3

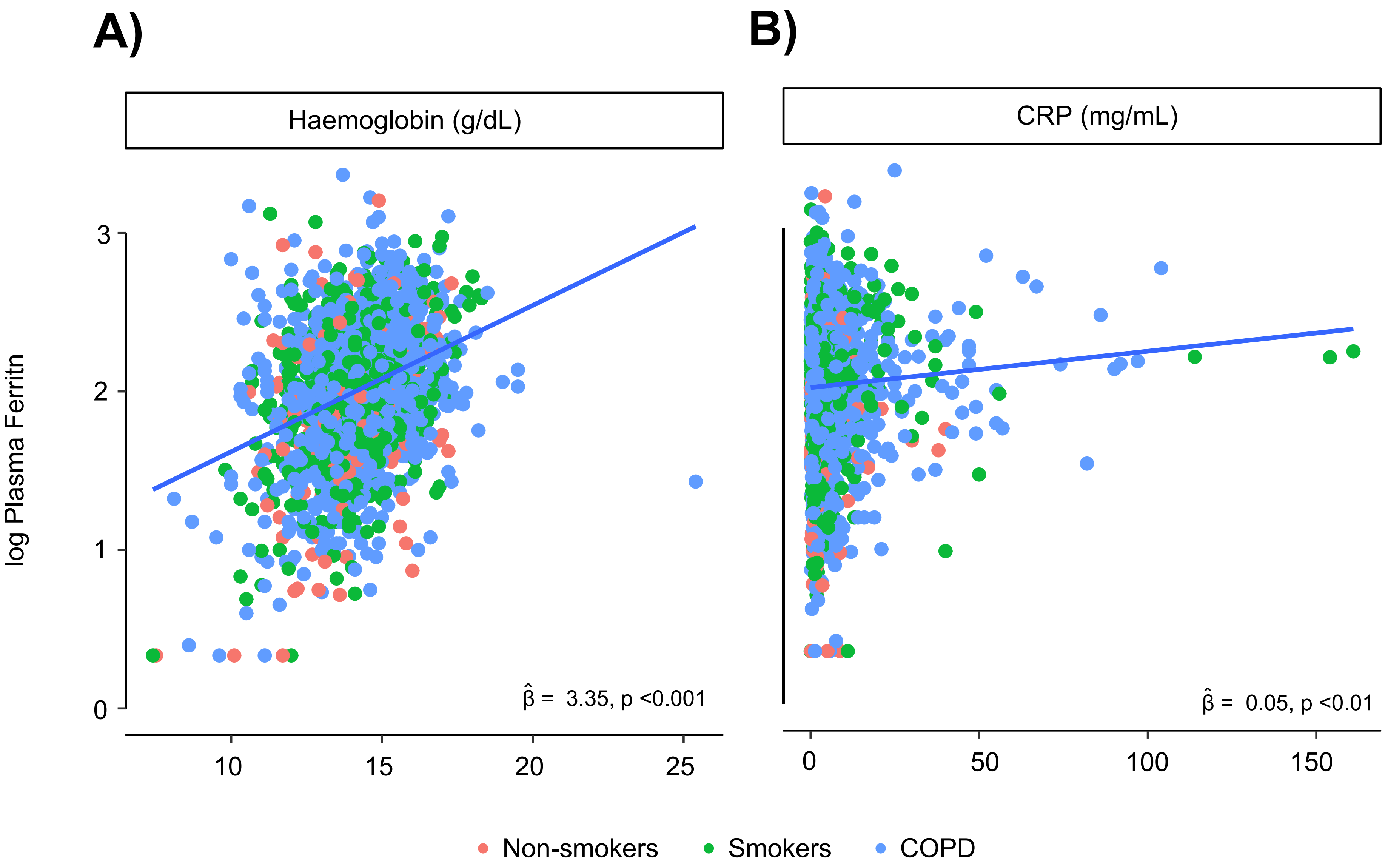

Supplemental Figure 4

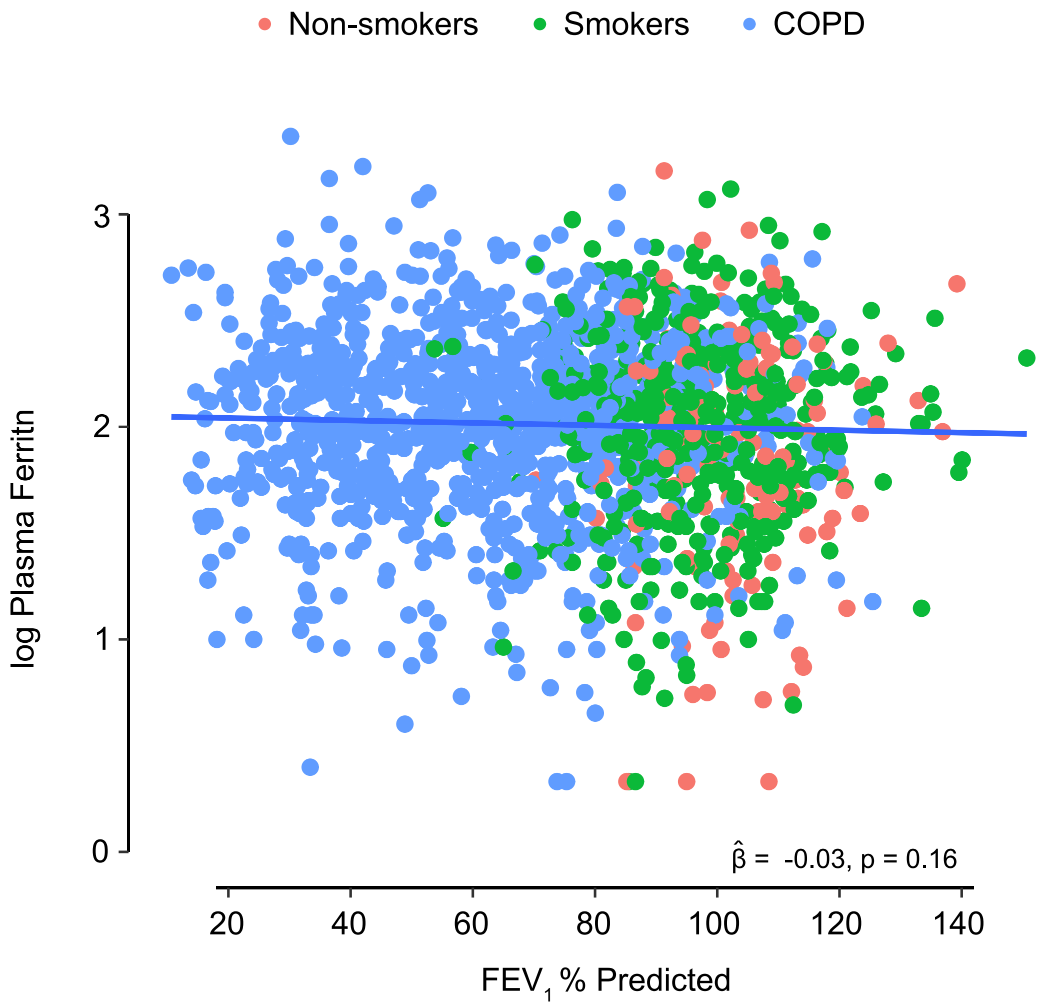

Supplemental Figure 5

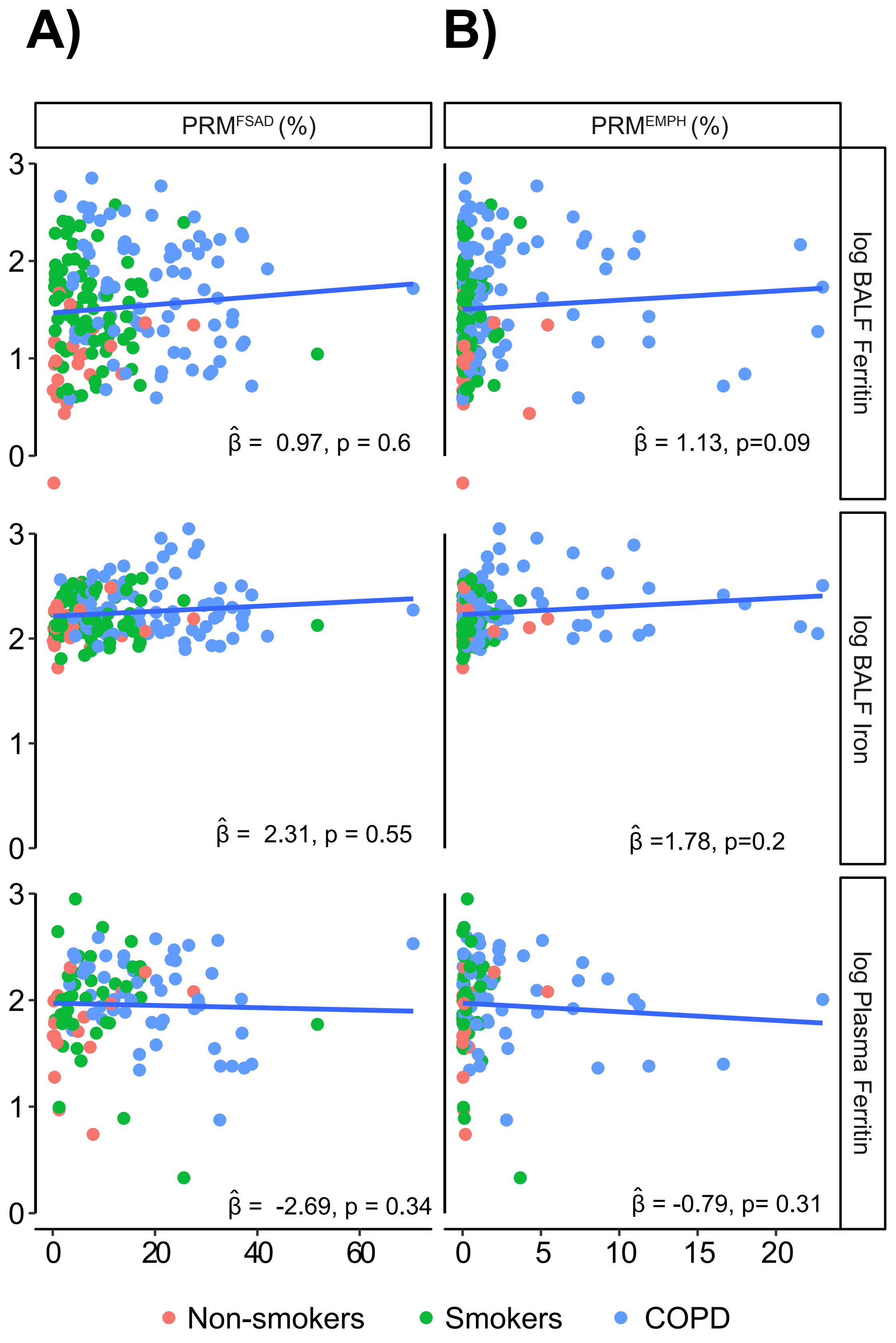

Supplemental Figure 6.
